# Non-alcoholic fatty liver disease is an influencing factor for the association of SHBG with metabolic syndrome in diabetes patients

**DOI:** 10.1038/s41598-017-15232-9

**Published:** 2017-11-06

**Authors:** Xiaomin Hua, Man Li, Fenghui Pan, Yunyun Xiao, Wenxia Cui, Yun Hu

**Affiliations:** 1 Department of Geriatrics, The Affiliated Drum Tower Hospital of Nanjing University, Nanjing, China; 2 Department of Cadre Health Care, The Qilu Hospital of Shandong University, Qingdao, China; 3 State Key Laboratory of Analytical Chemistry for Life Science Department of Chemistry Nanjing University, Nanjing, China

## Abstract

Metabolic syndrome (MS) and non-alcoholic fatty liver disease (NAFLD) have been identified as risk factors affecting serum sex hormone binding globulin (SHBG) levels. We conducted this cross-sectional study to delineate whether MS or NAFLD has more impact on circulating SHBG levels in type 2 diabetes (T2D) patients. Anthropometric and biochemical parameters including serums SHBG, testosterone (TT), liver enzymes, lipids, insulin, C-peptide and plasma glucose were measured. Regardless of the MS status, SHBG level was significantly lower in NAFLD patients than in non-NAFLD patients (*P* < 0.001). In the multiple linear regression analysis, lower serum SHBG level was strongly correlated with a higher incidence of NAFLD, but not MS components. In logistic regression analyses, after adjusted for age, sex, duration of diabetes, smoking status, and alcohol use, the ORs and 95%CI for presence of MS was 2.26 (95%CI 1.91–2.68) and for presence of NAFLD was 6.36 (95%CI 4.87–8.31) with per one SD decrease in serum SHBG (both P < 0.001). In conclusion, lower serum SHBG is associated with a higher prevalence of NAFLD, compared with MS and other metabolic disorders, in T2D patients. NAFLD might be an important influencing factor for the association of circulating SHBG with MS in T2D patients.

## Introduction

Sex hormone binding globulin (SHBG) is a glycoprotein made primarily in the liver^1^ and is well known as a transporter of sex steroids and regulates their bioavailability at tissue levels^2^. Epidemiological studies have found that serum SHBG level is altered in metabolic disorders, such as abdominal obesity, dyslipidemia, and insulin resistance^3,4^. Low serum SHBG has been considered as an independent predictive factor for metabolic syndrome (MS)^5,6^. However, several studies have highlighted the relationship between low SHBG with fatty liver. Our previous study has found that low serum SHBG, but not androgen, is independently associated with non-alcoholic fatty liver disease (NAFLD) in T2D patients^7^. A 9-month lifestyle interventional study has reported a significant correlation between magnetic resonance (MR) measured liver fat content and serum SHBG levels, but the relationship is independent of the changes in total body fat or abdominal fat mass^8^. Furthermore, a cross-sectional study has observed a significant association between the hepatic fat accumulation and circulating SHBG levels in men and premenopausal women, but no association between circulating SHBG and MS was observed^9^. However, whether MS or NAFLD has more impact on circulating SHBG levels has not been addressed.

MS and NAFLD share many metabolic abnormalities in glucose and lipid metabolism^10^. Subjects with MS are usually with a high presence of NAFLD than those without MS^11^. Meanwhile, many common metabolic disorders of MS and NAFLD have been identified as factors tightly related to SHBG^3,4^. Therefore, the association between serum SHBG, MS and NAFLD is complex.

A better elucidation for the association between serum SHBG with MS and NAFLD would help to understand their pathogenesis. Since SHBG is predominantly produced and regulated in the hepatocytes^12,13^ and its overexpression in NAFLD mouse models has been recently found to modulate hepatic lipogenesis^14^, we have reasoned that the apparent relationship between serum SHBG and MS may be mainly driven by the association of SHBG with NAFLD. Towards that end, we conducted this study aiming to delineate the relationship of serum SHBG with MS and NAFLD in T2D patients.

## Subjects and Methods

### Subjects and research design

This study was approved by the ethical committee of Nanjing Drum Tower hospital. Written informed consents were provided by all subjects prior to this study. All methods were performed in accordance with the ethical standards of the responsible committees for human experimentation and with the WMA Declaration of Helsinki. T2D patients who visited the Nanjing Drum Tower Hospital between September 2009 and December 2015 were sequentially enrolled into this retrospective study. T2D was defined based by World Health Organization (WHO) 1999 diagnostic criteria as fasting plasma glucose ≥7.0 mmol/l or 2-h plasma glucose ≥11.1 mmol/l ^15^. We excluded the subjects with acute or severe chronic diabetes complications, acute or chronic infections, hypo/hyperthyroidism, renal dysfunction, excessive alcohol consumption (men >30 g/day, women >20 g/day), autoimmune hepatitis or chronic viral liver disease, cholestasis or other metabolic/genetic liver disease, and the histories of taking corticosteroids, hormone, antiretroviral or anticancer drugs. A total of 1001 patients [608 males and 393 females; age: 58.5 ± 13.6 years-old (mean ± standard deviation (SD)), mean duration of diabetes: 8.6 ± 7.5 years] were enrolled into the study. Of them, 198 were newly diagnosed as T2D according to the WHO criteria, 425 received oral hypoglycemic medicines, 181 received insulin therapy and 197 received both medications.

### Diagnose of NAFLD

Diagnose of NAFLD was based on abdominal ultrasound performed by experienced ultra-sonographer after excluding other causes of chronic liver disease and excessive alcohol consumption as detailed above. Hepatic steatosis was diagnosed according to the following ultra-sonographic criteria: abnormal intensity and high-level echoes from the hepatic parenchyma, depths of echo penetration into of the liver, liver-kidney differences in echo amplitude and blurred liver vessel structure and abnormal visualization of diaphragm^16^.

### Definitions of metabolic syndrome

MS was diagnosed according to the 2005 IDF criteria^17^: (1) abdominal obesity (waist circumference ≥90 cm for Asian men or ≥80 cm for Asian women); (2) at least two of the following four components: 1) triglycerides ≥150 mg/dl (1.7 mmol/L), or specific treatment for this lipid abnormality; 2) HDL cholesterol <40 mg/dl (1.03 mmol/L) for men or <50 mg/dl (1.29 mmol/L) for women, or specific treatment for this lipid abnormality; 3) systolic/diastolic blood pressure ≥130/85 mmHg or receiving drug treatment; and 4) fasting plasma glucose ≥100 mg/dl (5.6 mmol/L), or previously diagnosed T2D or receiving treatment.

In order to determine the relationship of SHBG with the complexity of MS, we divided patients into five subgroups, MS1, MS2, MS3, MS4, and MS5, according to the number of MS components (MS score).

### Anthropometric and biochemical measurements

Anthropometric parameters of height, weight and waist circumference (WC) were measured using standard equipment and techniques. Blood was drawn in the morning after a 12-h overnight fast and was analyzed for alanine aminotransferase (ALT), aspartate aminotransferase (AST), gamma-glutamyl transpeptidase (GGT), total cholesterol (TC), triglycerides (TG), high density lipoprotein cholesterol (HDL-C), low density lipoprotein cholesterol (LDL-C), fasting plasma glucose (FPG, glucose oxidize technique method), glycosylated haemoglobin (HbA1c, high-performance liquid chromatography), fasting serum insulin (F-INS), fasting C-peptide (F-CP), total testosterone (TT), and serum SHBG (Siemens healthcare Diagnostics Products limited, Bad Nauheim, Germany) at the central clinical laboratory of the hospital. Body mass index (BMI) was calculated as weight (in kilograms) divided by the square of height (in meters). The insulin resistance was calculated using the homeostasis model assessment method (HOMA-IR = fasting insulin*fasting glucose/22.5)^18^.

### Statistical analysis

Continuous data were expressed as means ± SD or standard error of the mean (SEM). Categorical data were expressed as percentages. Variables not normally distributed were log-transformed before analyses. Continuous data were tested with independent samples t-test or Mann–Whitney U test, as appropriate. Categorical variables were tested with Pearson χ^2^-test. One-way analysis of variance (ANOVA) followed by Bonferroni post hoc test was used to compare the difference of SHBG between NAFLD and non-NAFLD groups and MS score subgroups. Relationships between SHBG and metabolic parameters were performed using partial correlation analysis adjusted for age and sex. Multiple linear regression models were conducted for the independent variables which significantly related to SHBG in univariate analysis before. The binary logistic regression analyses were used to determine the effects of decreasing serum SHBG levels on prevalence of MS and NAFLD. Potential confounding factors such as age, sex, duration of diabetes, smoking status, alcohol use, BMI, WC, GGT, TG, HDL-C, HOMA–IR and TT, which have been reported in researches to influence MS and NAFLD incidence were taken into consideration in different adjustment models. The results were expressed as odds ratios (OR) and 95% confidence intervals (CI). All statistical analyses were performed using SPSS 20.0 software (SPSS, Chicago, IL, USA). *P*-value of <0.05 was considered statistically significant.

### Data availability

The datasets generated during and/or analysed during the current study are available from the corresponding author on reasonable request.

## Results

### Baseline characteristics of T2D patients with and without NAFLD

According to ultrasonic diagnosis, all T2D patients were divided into NAFLD group (n = 453, age: 54.60 ± 12.92 years) and non-NAFLD group (n = 548, age: 61.75 ± 13.29 years). 71.9% (n = 326) patients were diagnosed with MS in NAFLD group, and 40.1% (n = 220) patients were diagnosed with MS in non-NAFLD group. Variables of BMI, WC, ALT, AST, GGT, TG, FPG, F-INS, fasting C-peptide, and HOMA-IR in the NAFLD group were significantly higher, compared with the non-NAFLD group (all *P* < 0.001). On the contrary, HDL-C in the NAFLD group was significantly lower than that in the non-NAFLD group (*P* < 0.001) **(**Table 1 **)**.

**Table 1:** Comparison of Baseline Characteristics between NAFLD and non-NAFLD groups.

	**non-NAFLD (n** = **548)**	**NAFLD (n** = **453)**	***P*** **value**
Sex (Male/Female)	306/242	302/151	0.001
MS (%)	220 (40.1%)	326 (71.9%)	<0.001
Age (year)	61.75 ± 13.29	54.60 ± 12.92	<0.001
Duration (year)*	10.30 ± 7.74	6.67 ± 6.79	0.005
BMI (kg/m^2^)	23.61 ± 2.86	26.57 ± 3.26	<0.001
WC (cm)	88.10 ± 9.27	95.60 ± 9.53	<0.001
ALT (U/L)*	20.16 ± 12.05	31.79 ± 22.38	<0.001
AST (U/L)*	19.34 ± 7.37	23.76 ± 13.22	<0.001
GGT (U/L)*	23.76 ± 19.53	39.18 ± 29.12	<0.001
TG (mmol/L)*	1.44 ± 1.17	2.47 ± 1.92	<0.001
TC (mmol/L)	4.43 ± 1.10	4.55 ± 1.20	0.106
HDL–C (mmol/L)	1.14 ± 0.33	0.94 ± 0.27	<0.001
LDL–C (mmol/L)	2.38 ± 0.79	2.35 ± 0.81	0.606
FPG (mmol/L)	8.18 ± 3.57	9.04 ± 3.56	<0.001
HbA1c (%)	9.28 ± 2.50	9.33 ± 2.02	0.723
F-INS (mU/L)*	9.28 ± 8.85	11.21 ± 9.30	<0.001
FCP (pmol/L)	602.99 ± 370.51	893.71 ± 443.84	<0.001
HOMA-IR	3.29 ± 1.37	4.45 ± 2.15	<0.001
SHBG (nmol/L)	42.54 ± 17.17	23.94 ± 10.64	<0.001
Male	39.60 ± 14.83	22.64 ± 9.67	<0.001
Female	46.25 ± 19.13	26.57 ± 11.97	<0.001

NAFLD, non-alcoholic fatty liver disease; MS, metabolic syndrome; BMI, body mass index; WC, waist circumstance; ALT, alanine aminotransferase; AST, aspartate aminotransferase; GGT, gamma-glutamyl transpeptidase; TG, triglyceride; TC, total cholesterol; HDL-C, high density lipoprotein cholesterol; LDL-C, low density lipoprotein cholesterol; FPG, fasting plasma glucose; HbA1c, glycosylated hemoglobin; F-INS, fasting insulin, F-CP, fasting C-peptide, HOMA-IR, homeostasis model assessment index of insulin resistance; SHBG, sex hormone-binding globulin. Date were presented as means ± standard deviation (SD) or proportion. Continuous data with normally distribution were tested by the independent samples t-test. *Continuous date without normally distribution were tested by the Mann–Whitney U test.

### Association of SHBG levels with MS components in patients with and without NAFLD

**Figure 1:**
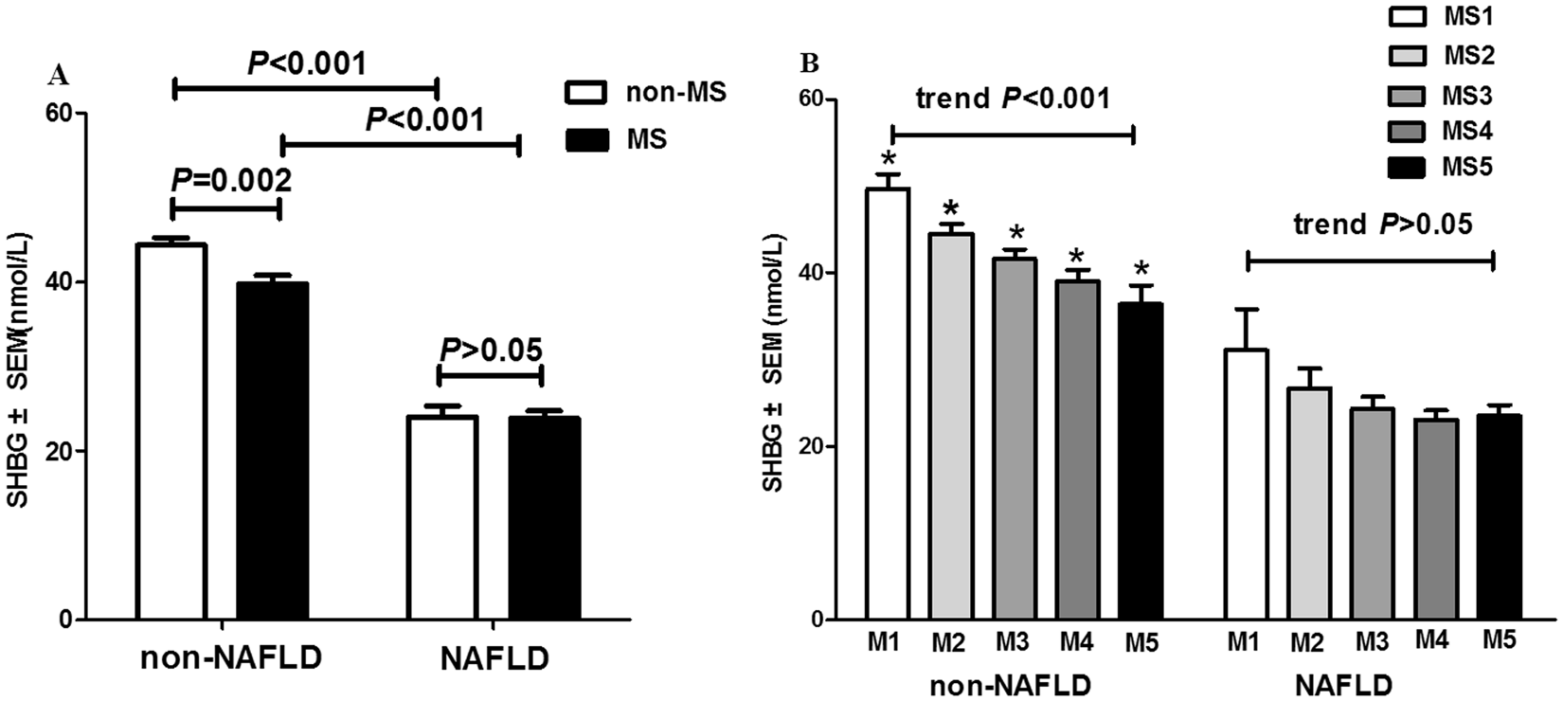
(**A**) Comparison of serum SHBG (sex hormone-binding globulin) in NAFLD (non-alcoholic fatty liver disease) and non-NAFLD groups, MS (metabolic syndrome) and non-MS subgroups. (**B**) Comparison of serum SHBG according to the MS score (number of MS components, M1-M5) between NAFLD and non-NAFLD groups. *P < 0.001, non-NAFLD *vs*. NAFLD. Linear trend in the mean level of SHBG by increased MS score in non-NAFLD group (trend *P* < 0.001, one-way analysis of variance (ANOVA)) and NAFLD group (trend *P* = 0.059, one-way ANOVA). Date were expressed as means ± standard error (SEM).

To determine the association of SHBG with MS status, we divided all study subjects into groups with or without MS and divided them into five subgroups from M1 to M5 according to the number of metabolic components. As shown in Fig. 1A, SHBG levels in NAFLD group were significantly lower than that in the non-NAFLD group, with ~45.8% and ~39.9% decline in non-MS and MS subgroups [non-MS group: 44.38 ± 0.80 *vs*. 24.04 ± 1.29 (mean ± SEM) nmol/L; MS group: 39.79 ± 0.98 *vs*. 23.91 ± 0.83 nmol/L; both *P* < 0.001]. However, the difference of serum SHBG levels between MS and non-MS subgroups was only statically significant in non-NAFLD group (*P* = 0.002), but not in NAFLD group (*P* > 0.05) **(**Fig. 1A **)**. This analysis shows that the NAFLD condition contributed more to the SHBG reduction (~43% decrease) in both non-MS and MS patients, whereas the MS status contributed to a less degree of SHBG reduction (~10.3%) in non-NAFLD patients **(**Fig. 1A **)**.

With the number of MS components increasing, serum SHBG levels decreased, with the linear trend statically significant in non-NAFLD group (*P* for linear trend <0.001, one-way ANOVA), but not in NAFLD group (*P* for linear trend = 0.059, one-way ANOVA). Mean levels of SHBG in each MS score subgroups of non-NAFLD were significantly higher than those in NAFLD (*P* < 0.001) **(**Fig. 1B **)**.

### Correlation of serum SHBG with metabolic variables and sex hormones

Correlation analyses showed that, after adjusted for age and sex, serum SHBG levels were negatively associated with BMI, WC, ALT, GGT, TG, FPG, F-INS, fasting C-peptide, HOMA-IR, and positively associated with HDL-C (*P* < 0.05), whereas serum SHBG levels were not related to AST, TC, LDL-C or HbA1c (*P* > 0.05) **(**Supplementary Table S1 **)**.

**Figure 2:**
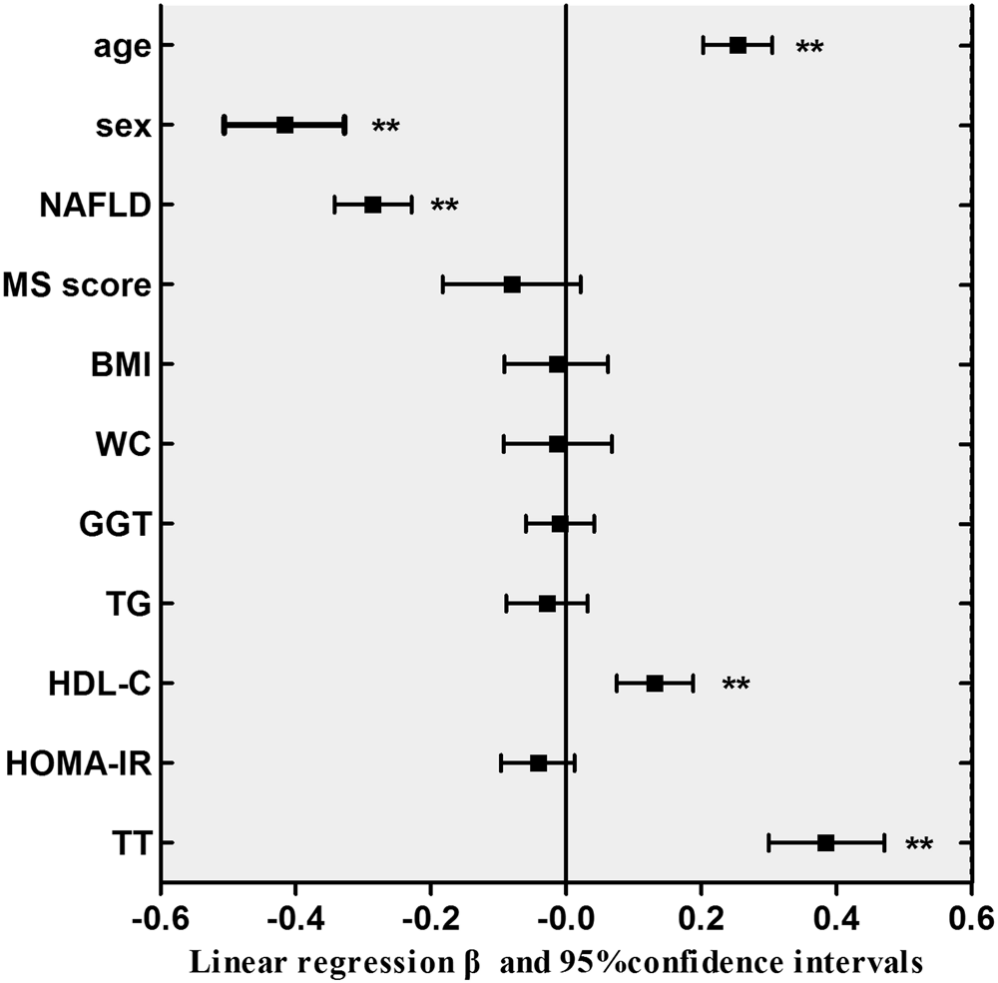
Multiple linear regression analysis between SHBG and metabolic variables. Dependent variable: SHBG (sex hormone-binding globulin). Independent variables: age, sex, NAFLD (non-alcoholic fatty liver disease), MS score (number of metabolic syndrome components), BMI (body mass index), WC (waist circumstance), GGT (gamma-glutamyl transpeptidase), TG (triglycerides), HDL-C (high density lipoprotein cholesterol), HOMA-IR (homeostasis model assessment index of insulin resistance), TT (total testosterone). β: standardized coefficients. *P < 0.05, **P < 0.01.

Factors found to be significantly associated with SHBG in the above correlation analysis were further analyzed for relative significance in multiple linear regression models. As a result, after adjustment for age, sex, BMI, WC, GGT, TG, HDL-C, HOMA-IR, and TT, the association between serum SHBG levels and MS scores reached a statistical significance with standardization coefficient (β) and 95% confidence interval (CI) was −0.165 (95%CI −0.241, −0.088) (*P* = 0.007). When NAFLD being added into this regression model as an independent variable, a strong correlation was found between lower serum SHBG levels and higher NAFLD incidence with β and 95%CI was −0.295 (95%CI −0.352, −0.239) (*P* < 0.001). Nevertheless, in this model, no independent relationship was found between the serum SHBG level and MS scores with β and 95%CI was −0.081 (95%CI −0.183, 0.021) (*P* = 0.057) (Fig. 2).

### Independent relationship between low serum SHBG and presence of MS/NAFLD

**Figure 3:**
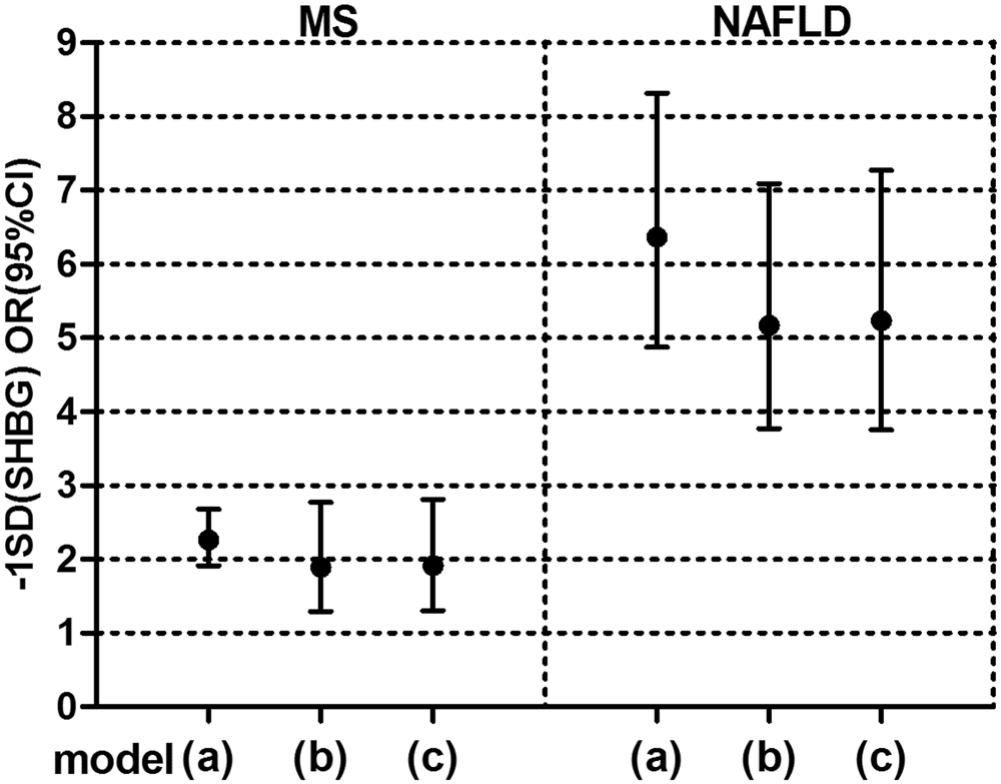
Decrease of SHBG has a larger impact on the presence of NAFLD than MS. The odds ratios (ORs) and 95% confidence intervals (CI) for presence of MS (metabolic syndrome) and NAFLD (non-alcoholic fatty liver disease) with per one standard deviation (SD) decrease in SHBG (sex hormone-binding globulin) (SD = 17.27 nmol/L). Model a: adjusted for age, sex, duration of diabetes (quartiles), smoking status, alcohol use; Model b: adjusted for a + BMI (body mass index), WC (waist circumstance), GGT (gamma-glutamyl transpeptidase, log-transformed), TG (triglycerides), HDL-C (high density lipoprotein cholesterol), HOMA–IR (homeostasis model assessment index of insulin resistance); Model c: adjusted for Model b + TT (total testosterone, log-transformed).

Binary logistic regression analyses were used to determine the effects of decreased serum SHBG levels on the incidence states of either MS or NAFLD. To illustrate the independent relationship between serum SHBG and MS/NAFLD, we conducted the following adjustment models to eliminate the potential confounders. In model a, after adjusted for age, sex, duration of diabetes (quartiles), smoking status, and alcohol use, the ORs and 95%CI for presence of MS was 2.26 (95%CI 1.91, 2.68) and for presence of NAFLD was 6.36 (95%CI 4.87, 8.31) with per one SD decrease in serum SHBG (SD = 17.27 nmol/L) (both *P* < 0.001). In model b, with further adjustment for BMI, WC, GGT (log-transformed), TG, HDL–C, and HOMA–IR, the SHBG related ORs for presence of both MS and NAFLD decreased slightly (*P* = 0.001 for MS, *P* < 0.001 for NAFLD). Moreover, the additional adjustment for total testosterone (log-transformed) in model c did not obviously influent the related ORs of Model b. The related ORs and 95%CI for MS was 1.91 (95%CI 1.30, 2.81) (*P* = 0.001) and for NAFLD was 5.23 (95%CI 3.75, 7.27) (*P* < 0.001) **(**Fig. 3 **)**. Thus, the decrease of SHBG exerts a larger impact on the presence of NAFLD than MS.

## Discussion

In the present study, we provide evidence that NAFLD is an influencing factor for the association of serum SHBG with MS in T2D patients. Firstly, low circulating SHBG is prevalent in NAFLD than in non-NAFLD patients, regardless of the MS status. Secondly, although circulating SHBG levels are associated with scores of MS in T2D patients, the association is no longer significant when NAFLD is included in the study. Thirdly, the decrease of circulating SHBG has a larger impact on the presence of NAFLD than MS.

Our finding is in agreement with a population-based cohort study by Flechtner-Mors *et al*.^9^. In their study, ultrasound diagnosed fatty liver was shown to exert a significant influence on serum SHBG concentrations in men and in premenopausal women, while no association between serum SHBG and metabolic syndrome was found. Besides, our data support that fatty liver is perhaps an influencing factor for the association of serum SHBG level with MS in that when the parameter of NAFLD was added into our linear regression model, the association turned to be no longer statistically significant. This clinical finding is also supported by the animal study by Selva *et al*.^19^ that SHBG production is down-regulated in fatty liver due to the reduced expression of HNF-4a.

We also provide evidence that SHBG is more closely associated with NAFLD than MS. Although the result showed a correlation between SHBG and MS status, we found that the NAFLD condition contributed more to the lower SHBG, for that even in patients with all the five MS components, serum SHBG level was still higher in non-NAFLD group than that in NAFLD group (Fig. 1). Furthermore, with *per* SD decrease of serum SHBG, the ORs for presence of NAFLD increased 5.1–6.3 folds, compared to 1.8–2.2 folds for MS. Association of SHBG with MS has been observed in many studies where NAFLD is not separated from MS^5,6^. Our study provides an explanation for the association in that large percentage of MS patients coexist with NAFLD and that NAFLD and MS share with impaired insulin sensitivity and lipid dyslipidemia^10,20^. Since the association is independent of many known confounding factors such as obesity, insulin resistance, and lipid disorders, it indicates that the association of SHBG with those metabolic traits is probably a consequence of NAFLD on SHBG levels (Fig. 2).

Several studies have highlighted the significance of testosterone deficiency in pathogenesis of hepatic steatosis^21,22^, but these studies have not assessed the effect of SHBG on this association. Our studies indicated that serum SHBG, but not testosterone, is associated with fatty liver or other metabolic disorders. The mechanism for the link between NAFLD and SHBG level remains to be further illustrated^23^. Saez-Lopez *et al*.^14^ recently showed that SHBG plays a suppressive role in hepatic lipogenesis in that SHBG overexpression in NAFLD mouse model ameliorates liver fat accumulation. Hence, there appears a vicious feedback between NAFLD and SHBG in the liver: fat accumulation suppresses SHBG production and the resulting lower SHBG results in fat accumulation. More studies in this respect may lead to new therapeutic approach for NAFLD.

## Conclusions

The current study shows that serum SHBG is significantly associated with metabolic disorders of abdominal obesity, liver enzyme, blood lipids, and insulin resistance. However, SHBG levels are significantly lower in NAFLD patients than in non-NAFLD patients regardless of the MS status. Lower serum SHBG is more associated with a higher prevalence of NAFLD, compared with MS and other metabolic disorders. Hence, the reduction of SHBG level in MS and T2D subjects is probably driven by fatty liver.

## Electronic supplementary material

Supplementary Table S1

## References

[1] Petra PH (1991). The plasma sex steroid binding protein (SBP or SHBG). A critical review of recent developments on the structure, molecular biology and function. J Steroid Biochem Mol Biol..

[2] Caldwell JD, Jirikowski GF (2009). Sex hormone binding globulin and aging. Horm Metab Res..

[3] Derby CA, Zilber S, Brambilla D, Morales KH, McKinlay JB (2006). Body mass index, waist circumference and waist to hip ratio and change in sex steroid hormones: the Massachusetts Male Ageing Study. Clin Endocrinol..

[4] Osuna JA, Gómez-Pérez R, Arata-Bellabarba G, Villaroel V (2006). Relationship between BMI, total testosterone, sex hormone-binding-globulin, leptin, insulin and insulin resistance in obese men. Arch Androl..

[5] Bhasin S (2011). Sex Hormone–Binding Globulin, but Not Testosterone, Is Associated Prospectively and Independently With Incident Metabolic Syndrome in Men The Framingham Heart Study. Diabetes Care..

[6] Laaksonen DE (2004). Testosterone and sex hormone–binding globulin predict the metabolic syndrome and diabetes in middle-aged men. Diabetes Care..

[7] Hua XM (2014). Low serum sex hormonebinding globulin is associated with nonalcoholic fatty liver disease in type 2 diabetic patients. Clin Endocrinol..

[8] Peter A (2010). Relationships of circulating sex hormone-binding globulin with metabolic traits in humans. Diabetes..

[9] Flechtner-Mors M (2014). Associations of fatty liver disease and other factors affecting serum SHBG concentrations: a population based study on 1657 subjects. Horm Metab Res..

[10] Marchesini G (2001). Nonalcoholic fatty liver disease a feature of the metabolic syndrome. Diabetes..

[11] Kotronen A, Westerbacka J, Bergholm R, Pietilainen KH, YkiJarvinen H (2007). Liver fat in the metabolic syndrome. J Clin Endocrinol Metab..

[12] Ja¨nne M, Hammond GL (1998). Hepatocyte Nuclear Factor-4 Controls Transcription from a TATA-less Human Sex Hormone-binding Globulin Gene Promoter. J Biol chem..

[13] Selva DM (2009). Peroxisome-proliferator receptor gamma represses hepatic sex hormone-binding globulin expression. Endocrinology..

[14] Saez-Lopez C (2017). Sex Hormone-Binding Globulin Reduction in Metabolic Disorders May Play a Role in NAFLD Development. Endocrinology..

[15] Alberti KG (1998). Definition, diagnosis and classification of diabetes mellitus and its complications. Part 1: diagnosis and classification of diabetes mellitus provisional report of a WHO consultation. Diabet Med..

[16] Saadeh S (2002). The utility of radiological imaging in nonalcoholic fatty liver disease. Gastroenterology..

[17] Alberti KG, Zimmet P, Shaw J (2005). The metabolic syndrome-a new worldwide definition. Lancet..

[18] Matthews DR (1985). Homeostasis model assessment: insulin resistance and beta-cell function from fasting plasma glucose and insulin concentrations in man. Diabetologia..

[19] Selva DM, Hogeveen KN, Innis SM, Hammond GL (2007). Monosaccharide-induced lipogenesis regulates the human hepatic sex hormone-binding globulin gene. J Clin Invest..

[20] Kogisoa T, Moriyoshi Y, Nagahara H (2007). Clinical significance of fatty liver associated with metabolic syndrome. Hepatol Res..

[21] Kim S (2012). A low level of serum total testosterone is independently associated with nonalcoholic fatty liver disease. BMC Gastroenterol..

[22] Volzke H (2010). Hepatic steatosis is associated with low serum testosterone and high serum DHEAS levels in men. Int J Androl..

[23] Bonnet F (2013). Role of sex steroids, intra-hepatic fat and liver enzymes in the association between SHBG and metabolic features. Clin Endocrinol..

