# Supplementary material for: Non-alcoholic fatty liver disease is an influencing factor for the association of SHBG with metabolic syndrome in diabetes patients
Source: Sci Rep. 2017 Nov 6;7:14532. doi: 10.1038/s41598-017-15232-9 (PMC5674048; doi:10.1038/s41598-017-15232-9)
Supplement: Supplementary file 1 — Supplementary Table S1 [file 41598_2017_15232_MOESM1_ESM.doc]

**Non**-**alcoholic fatty liver disease is an influencing factor for the association of SHBG with metabolic syndrome in diabetes patients**

Xiaomin Hua^1,2*^, Man Li^1*^, Fenghui Pan^1^, Yunyun Xiao^1^, Wenxia Cui^1^, Yun Hu^1,3^

| **Factors** | **SHBG (nmol/L)** | |
| --- | --- | --- |
|  | **Partial r** | ***P*** |
| BMI(kg/m^2^) | **–**0.297 | <0.001 |
| WC(cm) | **–**0.310 | <0.001 |
| ALT(U/L) | **–**0.117 | <0.001 |
| AST(U/L) | **–**0.046 | 0.169 |
| GGT(U/L) * | **–**0.256 | <0.001 |
| TG(mmol/L) | **–**0.282 | <0.001 |
| TC(mmol/L) | 0.025 | 0.461 |
| HDL–C(mmol/L) | 0.328 | <0.001 |
| LDL–C(mmol/L) | 0.023 | 0.489 |
| FPG(mmol/L) | **–**0.111 | 0.001 |
| HbA1c (%) | 0.035 | 0.289 |
| F-INS(mU/L) * | **–**0.229 | <0.001 |
| F–CP(pmol/L) | **–**0.305 | <0.001 |
| HOMA–IR | **–**0.272 | <0.001 |

**Supplementary Table S1. Correlation of serum SHBG with metabolic variables and sex hormones.** SHBG, sex hormone-binding globulin; BMI, body mass index; WC, waist circumstance; ALT, alanine aminotransferase; AST, aspartate aminotransferase; GGT, gamma-glutamyl transpeptidase; TG, triglyceride; TC, total cholesterol; HDL-C, high density lipoprotein cholesterol; LDL-C, low density lipoprotein cholesterol; FPG, fasting plasma glucose; HbA1c, glycosylated hemoglobin; F-INS, fasting insulin, F-CP, fasting C-peptide, HOMA-IR, homeostasis model assessment index of insulin resistance. Partial correlation analyses adjusted for age and sex were performed. * log-transformed before analysis.
